# Who has the greatest influence on adolescent gaming disorder: parents, teachers, or peers? An interpersonal relationships network model of gaming disorder

**DOI:** 10.3389/fpsyt.2024.1419014

**Published:** 2024-10-07

**Authors:** Xinjie Tan, Chunlin Liu, WanJun Yang, Xiao Hui, Ling Zhang, Shuanghong Chen, Ying He

**Affiliations:** ^1^ Department of Medical Psychology, Neurological Medical Center, Xinqiao Hospital and The Second Affiliated Hospital, Army Medical University, Chongqing, China; ^2^ Department of Military Developmental Psychology, Faculty of Medical Psychology, Army Medical University, Chongqing, China

**Keywords:** network analysis, gaming disorder in adolescents, parent-child relationships, teacher-student relationships, peer relationships, interpersonal relationships

## Abstract

**Introduction:**

Gaming disorder (GD) in adolescents is associated with impaired interpersonal relationships, including those with parents, teachers and peers. However, the interpersonal relationships most strongly associated with GD-related maladaptive behaviors are not well established. This study aimed to investigate the associations between these three types of relationships and the manifestation of GD in adolescents.

**Methods:**

In this cross-sectional study, 1920 Chinese adolescents participated in a survey that assessed interpersonal relationships (parent−child, teacher−student, and peer relationships) and demographic variables (e.g., gender, grade, duration of gaming), and 1414 participants were ultimately included. A network analysis approach was utilized to evaluate the key network metrics of edge weight and node centrality.

**Results:**

The findings revealed that peer fear and inferiority (r = 0.12) and teacher−student conflict were most strongly correlated with GD, followed by parent−child conflict (r = 0.09). Peer fear and inferiority exhibited the highest strength centrality (0.84), followed by teacher−student conflict (0.83) and parent−child conflict (0.35). Moreover, the duration of gaming was significantly and positively correlated with GD (r = 0.19).

**Conclusions:**

The present study underscores the significant role of conflict and rejection within interpersonal relationships, particularly among peers, in the manifestation of GD-related behaviors in Chinese adolescents.

## 1 Introduction

Gaming disorder (GD), commonly referred to as gaming addiction, is officially included in the 11th revision of the International Classification of Diseases (ICD-11) and is classified as a substance use and addictive disorder (1). Presently, GD has emerged as a pervasive global concern, with a prevalence rate of 3.3% globally (2). The prevalence rate of GD has reached 9.9% (3) among young individuals worldwide, while it reached 17% in China (4), with a percentage as high as 23.83% among adolescents from ethnic minorities (5).

Individuals diagnosed with GD commonly exhibit a deficiency in outdoor activities, prolonged screen exposure resulting in joint and muscle pain (6), headaches (7), disrupted circadian rhythms, and imbalanced dietary patterns, leading to endocrine disruptions and compromised immunity (8). Individuals with GD frequently manifest diminished cognitive functions (9), heightened negative emotions (10), and a greater frequency of problematic behaviors (11). Furthermore, research indicates that heightened adverse effects on adolescents in crucial developmental stages are linked to GD. These effects include diminished academic performance, involvement in problematic behaviors such as theft and gambling, access to explicit content, and engagement in aggressive conduct (12, 13). Hence, GD among adolescents deserves greater attention.

Interpersonal factors play a pivotal role in comprehending and addressing issues related to adolescent GD (14, 15). Adolescents are experiencing a phase of development that involves significant changes in interpersonal relationships (e.g., emotional separation from parents, an increased sense of peer identification, and new attitudes toward teachers) (15). When experiencing maladaptive interpersonal behaviors, adolescents develop feelings of loneliness, which may lead to the development of GD (15, 16). Three types of interpersonal relationships are primarily involved here: parent−child relationships, teacher−student relationships, and peer relationships (14). Many studies have confirmed that unfavorable parent−child relationships, including parental rejection (17) and inadequate parental supervision (18), constitute risk factors for GD. Longitudinal evidence suggests that teacher autonomy support mitigates the severity of adolescent GD (19). Positive teacher−student relationships foster healthy growth and development in adolescents, reducing their addiction risks (20). Conversely, negative teacher−student relationships, including instances of teacher abuse, may contribute to GD in adolescents (14). Peer relationships also serve as crucial determinants of adolescent GD. Prior research has revealed a strong association between peer factors and GD, with deviant peer affiliation (21) and peer victimization (22) being closely linked to GD. Longitudinal studies additionally demonstrate that positive peer attachment negatively predicts individual GD behavior (23).

Ecological systems theory posits that family and school serve as two microsystems that directly influence adolescent development and exert the most direct and potent impact on adolescents (24). While prior studies have confirmed an intricate association between parent–child, teacher–student, and peer relationships and GD (14), the significance of their potential impact on GD remains infrequently discussed. Which of the three relationships above is most closely associated with GD remains unclear. Network analysis, an emerging method grounded in the system network model (25), enables simultaneous analysis of multiple influencing factors. In a network, nodes represent measured variables, and edges connecting the nodes signify correlations between two nodes after considering all other nodes in the control network. Network analysis can reveal genuine interactions between two nodes, mitigating the risk of spurious correlations resulting from the shared third variable (26). Non-zero edges in the network can signify mediations and potential causal paths (25), aiding in identifying the roles of variables within the network model and providing a more comprehensive explanation of event occurrences.

Network analysis methods have been utilized by researchers to explore the connections between interpersonal relationships and specific issues. Ge and Zhang (27) applied network analysis to compare the degree of association between parent-child, teacher-student, and peer relationships with adolescent suicidal ideation. Monteleone et al. (28) explored the relationships among interpersonal problems, emotion regulation, and eating symptoms in individuals with obesity using a network model. This demonstrates the potential of network analysis in addressing themes related to interpersonal relationships.

Therefore, this study aims to investigate the links between parent-child, teacher-student, and peer relationships and the problematic behaviors associated with gaming disorder (GD) in Chinese adolescents through network analysis, and to assess the relative strength of these relationships’ association with GD. The objective is to offer insights into the prevention and intervention strategies for adolescent gaming disorder.

## 2 Materials and methods

### 2.1 Participants

This study utilized a convenience cluster sampling method for a cross-sectional survey of 1,920 adolescents across four secondary schools in Sichuan Province and Chongqing Municipality, China. Because some students at the surveyed schools did not have access to mobile phones, the questionnaire survey was administered through a combination of offline and online surveys (using the Wen Juan Xin platform), which were conducted between January and February 2024. Informed consent was obtained from both the participants and their homeroom teachers for each survey. Adolescents in junior or senior high school with the ability to independently complete the survey were included in the study. Invalid questionnaires were excluded if they met the following criteria: (1) no gaming behaviors within the past year, (2) more than 90% of the responses were the same option, or (3) missing values were present. Ultimately, a total of 1414 valid questionnaires were included, for a response rate of 73.6%. This research was approved by the Ethics Committee of the Second Affiliated Hospital of the Army Medical University (Approval Number: 2023-Research No. 145-02).

### 2.2 Measures

#### 2.2.1 Demographics

Demographic information of the participants, including gender, grade, student origin, ethnic group, leadership status within the class, and weekly gaming hours, was gathered.

#### 2.2.2 Network of relationships inventory

We used the NRI, which is composed of a total of 30 items rated on a 5-point scale ranging from 1 (never) to 5 (always), to assess parent−child relationships (29). The dimensions of the questionnaire include Companionship, Instrumental Aid, Support, Intimacy, and Conflict. The Chinese version of the NRI was validated by Tian and Zhang (30). For this study, the Cronbach’s α coefficient for the overall scale was 0.92. Additionally, the Cronbach’s α coefficients for the dimensions of companionship, instrumental aid, support, intimacy, and conflict were 0.75, 0.80, 0.86, 0.83, and 0.85, respectively.

#### 2.2.3 Chinese peer relationship scale

The CPRS, composed of two subscales (Peer Acceptance and Peer Fear and Inferiority), was used to assess the peer relationships of the adolescents (31). It consists of a total of 30 items, utilizing a 4-point scale ranging from 1 (not at all) to 4 (very much). A higher score on the Peer Acceptance Scale indicates a greater degree of peer acceptance, and a higher score on the Peer Fear and Inferiority scale reflects elevated peer fear and inferiority, correlating with an increased likelihood of rejection by peers in communication. In this study, the Cronbach’s α coefficients for peer acceptance and peer fear and inferiority were 0.93 and 0.92, respectively.

#### 2.2.4 Chinese teacher–student relationship scale

The CSTRS was employed to assess the relationships between adolescents and their teachers (32) and contains a total of 23 items, utilizing a 5-point scale ranging from 1 (Did not apply to me at all) to 5 (Applied to me very much). The scale contains four dimensions: Satisfaction, Support, Conflict, and Intimacy. For this study, the Cronbach’s α coefficient for the overall scale was 0.91. Additionally, the Cronbach’s α coefficients for the dimensions of satisfaction, support, conflict, and intimacy were 0.71, 0.80, 0.82, and 0.84, respectively.

#### 2.2.5 Gaming disorder scale for adolescents

Following the ICD-11 diagnostic criteria for GD, Paschke et al. (41) developed an assessment tool specific to adolescent GD—the Adolescent Gaming Disorder Scale (GADIS-A). The GADIS-A consists of 10 items, utilizing a 5-point scale ranging from 0 (strongly disagree) to 4 (strongly agree). The presence of GD is indicated when the factor one score exceeds 5 points, the factor two score exceeds 9 points, and the time-frequency score surpasses 2 points. The Chinese version of the scale has good reliability and validity (40). For this study, the Cronbach’s α coefficient for the overall scale was 0.87.

### 2.3 Statistical analyses

Preliminary data analysis was performed using the statistical software SPSS (version 26.0), involving calculations for each node and an examination of Spearman correlations among all study variables. There were no missing data in the survey, as participants, upon consent, were prompted to complete all the items. In this study, participants self-reported all research variables. To address potential bias, we conducted Harman’s common method bias test, which involved using unrotated principal component analysis to examine the results of factor analysis. Four factors were extracted, and the maximum common factor explained 37.57% of the variance, which was below the critical value of 40%, indicating that common method bias was absent.

Subsequently, we constructed correlation matrices and network models using R software (version 4.3.1). We used Spearman correlations, instead of polychoric correlations, as inputs for network analysis to avoid potential bias caused by low frequencies between items in the marginal crosstables, which could affect the estimated polychoric correlations and subsequent partial correlations (25, 33). We employed a Gaussian graphical model (GGM) to establish the network between interpersonal relationships and gaming disorder variables. To reduce the number of connections in the network, we utilized the graphic least absolute shrinkage and selection operator (GLASSO) algorithm. In this model (i.e., GGM), nodes represent observed variables (i.e., the scores of scales or subscales used in this study), and edges represent the connection between two nodes while controlling for all other nodes. The blue lines indicate positive correlations, and the red lines indicate negative correlations. The thickness of the edges represents the strength of the correlation, described as edge weight terms (i.e., partial correlation coefficients ranging from -1 to 1). An absolute value of 0.03 or higher was deemed interpretable (34). We then used the qgraph package in R to visualize the network model. The mgm function calculates the node predictability index of the network, and the centralityPlot function is used to calculate centrality indices, including node strength centrality, betweenness centrality, and closeness centrality (25). These three centrality indices indicate the relative importance of a given node in the network, with higher values denoting greater centrality. Finally, for network accuracy and stability, we used the bootnet package. Following Epskamp et al. (25)’s guidelines, we first estimated the accuracy of edge weights by bootstrapping with 95% confidence intervals (CIs), with narrower CIs indicating a more precise estimation of the edge. Second, we assessed the stability of centrality indices by using the case-dropping bootstrapping. The stability examines whether the order of centrality indices remains the same after re-estimating the network with fewer cases or nodes. The correlation stability coefficient (CS-coefficient) is used to quantify the stability. This coefficient should not be below 0.25, and preferably above 0.5. Third, we used boot­strapped difference tests to examine the potential differences between edge-weights and centrality indices.

## 3 Results

### 3.1 Descriptive statistics and correlation analysis

In this study, a total of 1414 adolescents were included for analysis, with 775 (54.8%) boys and 639 (45.2%) girls. Among them, 781 (55.2%) were middle school students, while 633 (44.8%) were high school students. Geographically, 605 (42.8%) students were from urban areas, and 807 (57.2%) were from rural areas. Regarding ethnic composition, the majority were Han Chinese, totaling 1,374 (97.2%), with 40 individuals (2.8%) from ethnic minorities. In terms of leadership roles, 621 participants (43.9%) held class cadre positions, whereas 793 participants (56.1%) did not hold such positions.

**Figure 1:**
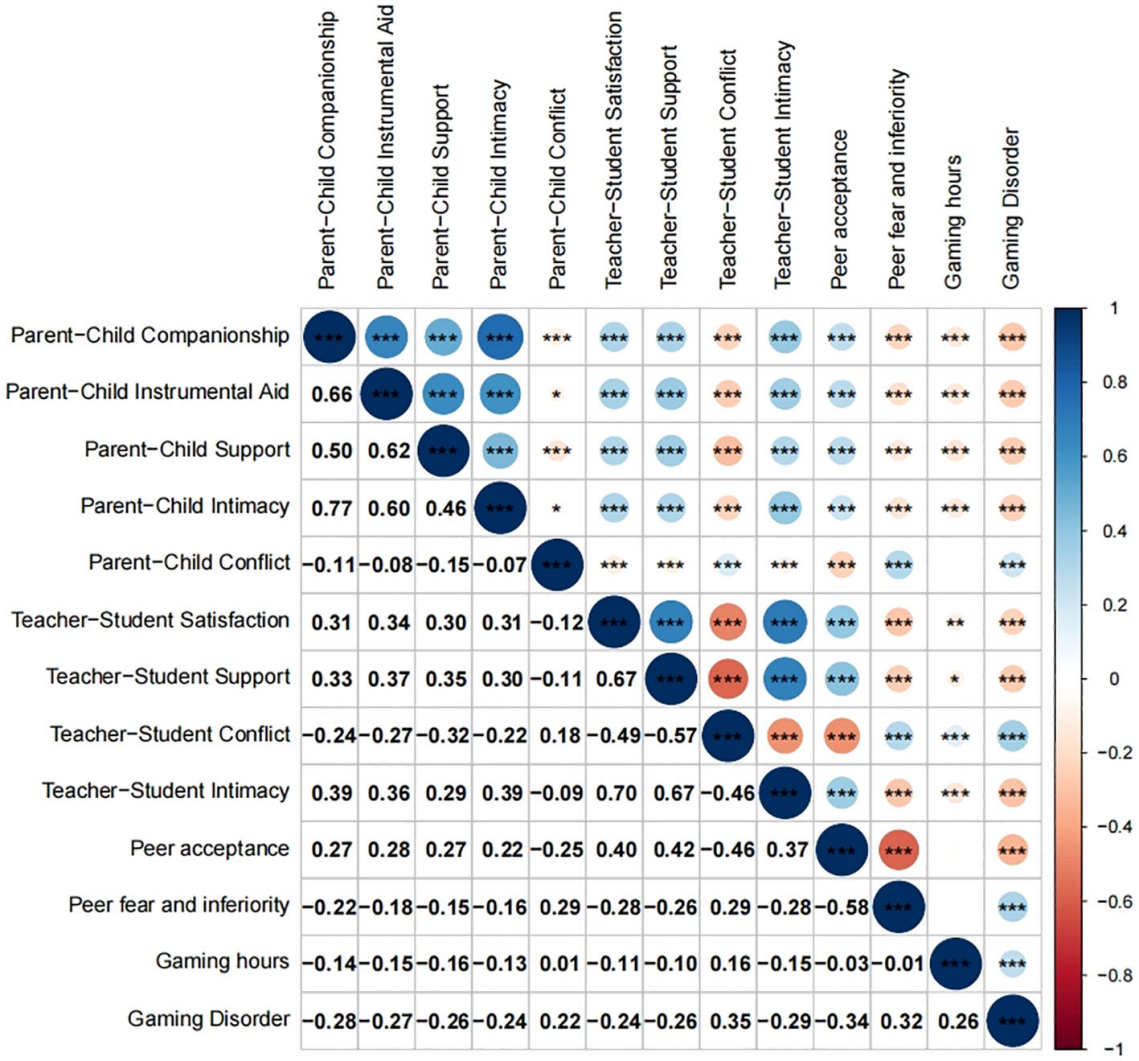
Correlation matrix heat map for study variables. The numbers in the lower triangle matrix are the Spearman correlation coefficient in the upper triangle matrix, and the blank box represents *p* > 0.05 corresponding to the correlation coefficient. “*”, “**” and “***” represent *p* < 0.05, *p* < 0.01 and *p* < 0.001 respectively.

**Table 1** displays the mean, standard deviation, and range of all variables in this study. As shown in **Figure 1**, the severity of GD was negatively correlated with parent–child companionship, instrumental help, emotional support, intimacy, teacher–student satisfaction, teacher–student support, teacher–student intimacy, and peer acceptance (p < 0.001). Conversely, GD scores were positively correlated with parent−child conflict, teacher−student conflict, peer fear and inferiority, and playtime (p < 0.001).

**Table 1:** Mean scores and standard deviations of all variables.

Variable	Mean	SD	range
Parent-Child Relationships			
Companionship	19.11	4.73	6-30
Instrumental Aid	22.88	4.74	6-30
Support	26.05	4.30	6-30
Intimacy	17.48	5.41	6-30
Conflict	14.67	4.64	6-30
Teacher-Student Relationships			
Satisfaction	18.88	3.53	5-25
Support	16.47	2.88	4-20
Conflict	12.35	4.69	7-35
Intimacy	24.40	5.42	7-35
Peer Relationships			
Peer acceptance	65.00	10.24	26-80
peer fear and inferiority	20.80	7.30	1-40
Gaming Disorder			
Gaming hours	4.47	6.10	0.1-23
Gaming Disorder	9.26	6.12	0-36

### 3.2 Network analysis

**Figure 2:**
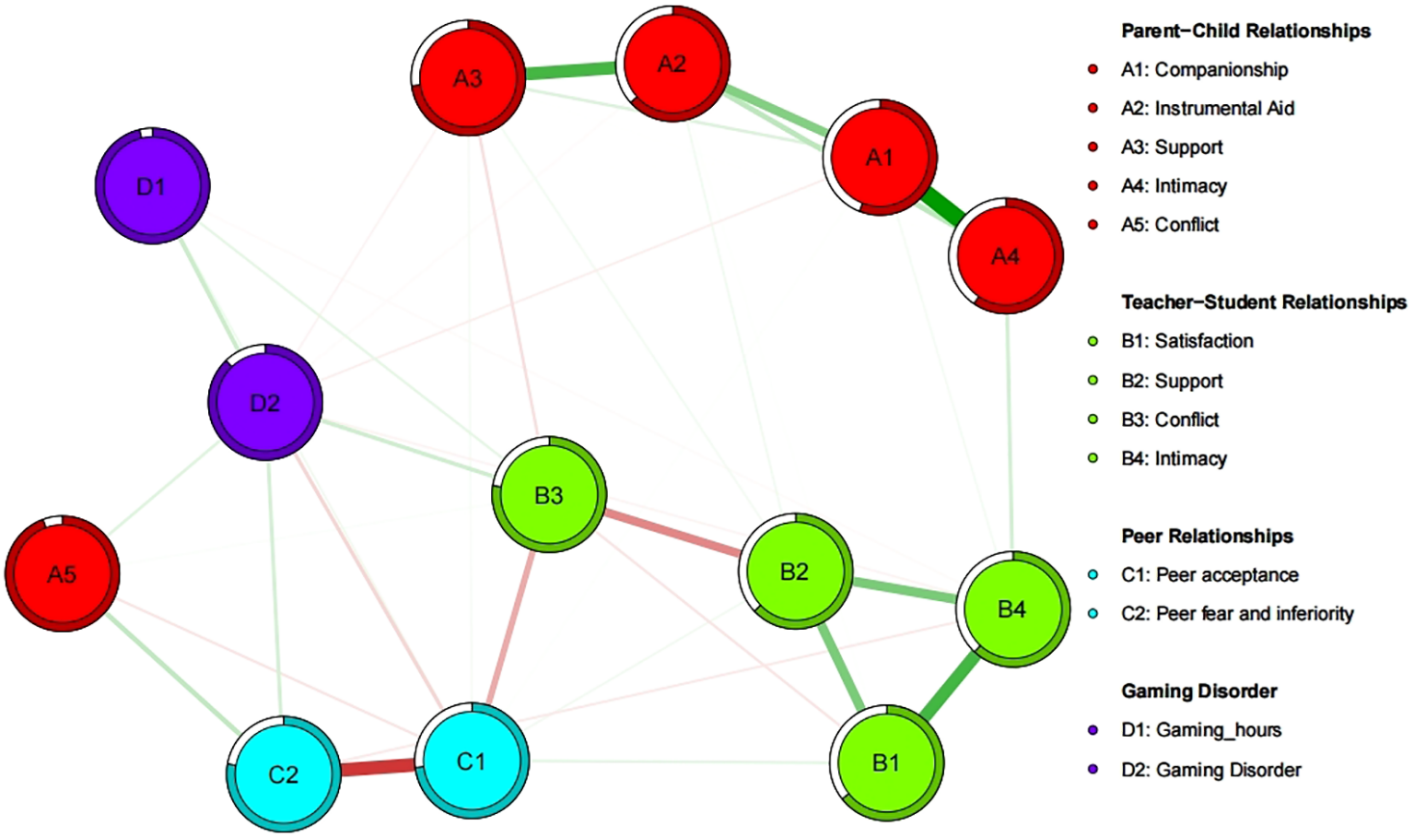
Network model of interpersonal relationships and gaming disorder. Network model of interpersonal relationship and gaming disorder composed of 13 variables. Each variable is represented by a node (A1-D2). The green line is a positive connection and the red line is a negative connection. The thickness of the line represents the strength of the connection. The blank area in the ring around the node represents the predictability of the node (the percentage of variance of the current node as interpreted by the surrounding nodes).

**Figure 2** presents a network model illustrating the interplay between interpersonal relationships and GD involving 13 nodes (A1-D2). Among all nonzero edges related to GD (D2), the most robust positive correlation was observed between GD (D2) and gaming hours (D1) (r = 0.19). Among the interpersonal variables, GD (D2) exhibited the strongest associations with peer fear and inferiority (C2), teacher−student conflict (B3), and parent−child conflict (A5) (r = 0.12, r = 0.12, r = 0.09). Conversely, GD (D2) showed strong negative associations with peer acceptance (C1) and parent−child companionship (A1) (r = -0.08, r = -0.06). The edges linked to teacher−student intimacy (B4), parent−child instrumental aid (A2), and parent−child support (A3) exhibited weaker associations (r = -0.04, r = -0.03, r = -0.03).

The estimated stability coefficient (CS) of the network revealed that the stability coefficient for strength centrality was optimal, measuring 0.75 and exceeding 0.5, indicating that the stability of the network estimation results was satisfactory. Therefore, this study primarily relies on strength centrality to assess the significance of each node in the network.

**Figure 3:**
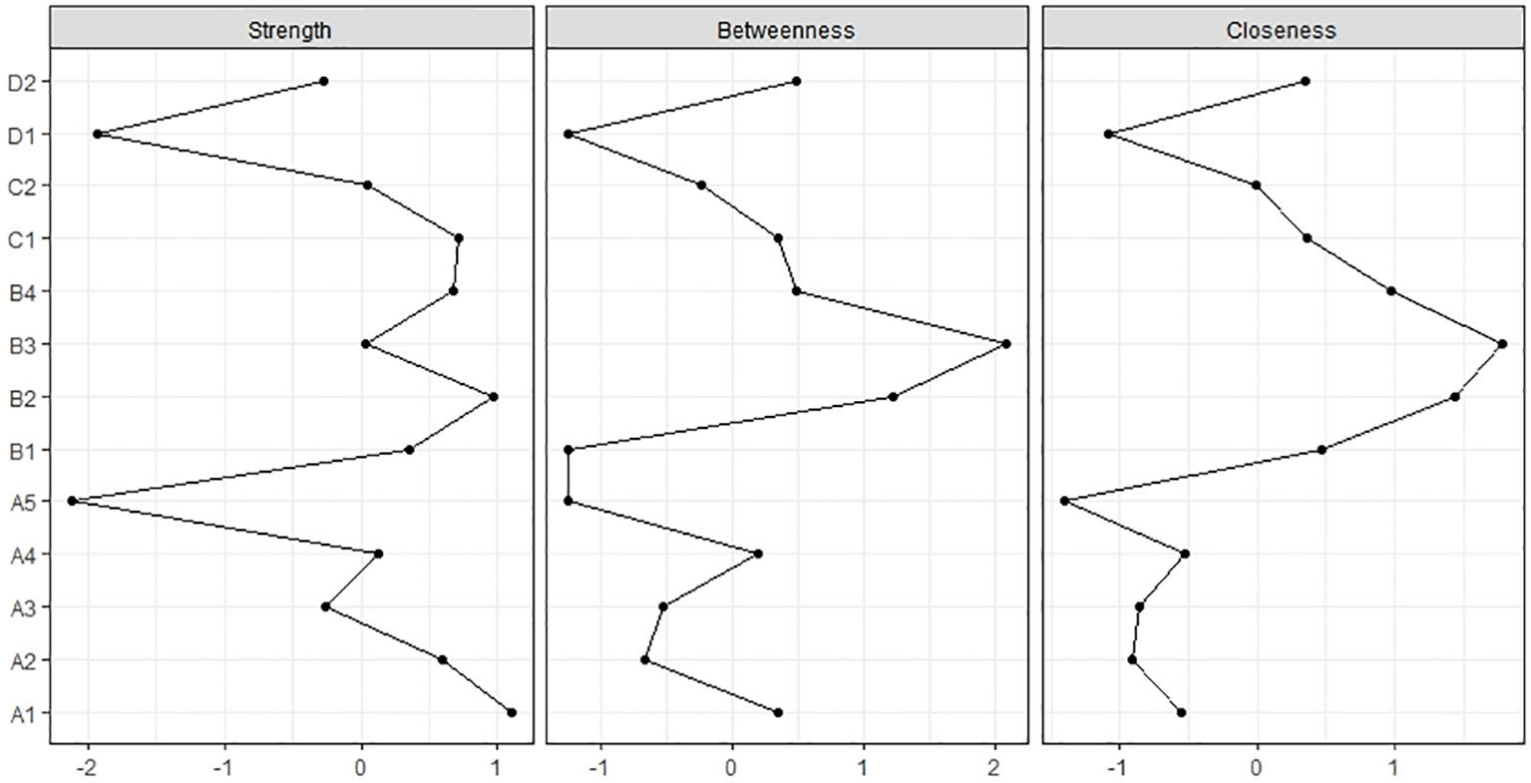
Centrality plots of network analysis. Centrality plots for the 13 nodes depicted as strength, betweenness, and closeness. The X-axis represents standardized z-scores of these three centrality indices (the higher the value, the more central the node), and the Y-axis represents the 13 variables.

As shown in **Table 2** and **Figure 3**, the strength centrality of peer fear and inferiority (C2), teacher−student conflict (B3), and parent−child conflict (A5), which exhibited the strongest associations with GD (D2), were 0.84, 0.83, and 0.35, respectively. Notably, peer fear and inferiority (C2) demonstrated greater centrality than teacher−student conflict (B3) and parent−child conflict (A5).

**Table 2:** List of nodes, their predictability, and their centrality estimation.

Nodes	Variables	Predictability	Centrality		
			Strength	Betweenness	Closeness
A1	Companionship	0.69	1.07	11.00	0.01
A2	Instrumental Aid	0.60	0.96	4.00	0.00
A3	Support	0.47	0.77	5.00	0.00
A4	Intimacy	0.65	0.85	10.00	0.01
A5	Conflict	0.11	0.35	0.00	0.00
B1	Satisfaction	0.59	0.90	0.00	0.01
B2	Support	0.60	1.04	17.00	0.01
B3	Conflict	0.40	0.83	23.00	0.01
B4	Intimacy	0.62	0.98	12.00	0.01
C1	Peer acceptance	0.47	0.99	11.00	0.01
C2	peer fear and inferiority	0.39	0.84	7.00	0.01
D1	Gaming hours	0.07	0.39	0.00	0.00
D2	Gaming Disorder	0.23	0.76	12.00	0.01

Predictability, which represents the rate at which a node’s variation is explained by nodes connected to it in the network. Strength centrality is an indicator that estimates the importance of a node. The higher the node, the more important the node is in the network. Intermediary centrality, which estimates the degree of influence exerted by other nodes through this node. A higher node indicates that the other nodes in the network have more connections through it. Proximity centrality, the inverse of the sum of the shortest path lengths from this node to other nodes in the network, with higher nodes indicating a greater likelihood of being rapidly affected by changes in the remaining nodes.

Additionally, the predictability of GD (D2) was 0.23, indicating that 23% of the variance in node D2 can be explained by its surrounding nodes. Across the network, the average node predictability was 0.45, suggesting that, on average, 45% of the variance of each node can be explained by the connected nodes in this network model.

## 4 Discussion

The network analysis revealed a close association between the dimensions of conflict and rejection within the three types of interpersonal relationships and GD. Specifically, peer fear and inferiority, teacher−student conflict, and parent−child conflict exhibit the strongest correlations with GD, with peer fear and inferiority having the highest strength centrality among the three. This suggests that, in comparison to teacher−student conflict and parent−child conflict, peer fear and inferiority play a more prominent role in the interaction between interpersonal relationships and GD, demonstrating a closer association with GD. A higher level of fear and inferiority corresponds to an increased risk of GD, aligning with findings of previous studies (22). According to Bussone and colleagues (35), in instances of peer bullying, online games serve as an effective means for individuals to escape from reality. Simultaneously, they offer a safe environment for individuals to forge interpersonal relationships in the virtual world, compensating for deficiencies in real-life connections and thereby increasing individuals’ susceptibility to GD. This implies that addressing peer communication challenges among adolescents in real life should be one of the foremost targets of psychological intervention.

On the other hand, within the network, the connection weight and strength centrality of teacher−student conflict and GD rank second only to peer fear and inferiority. This underscores the impact of teacher−student relationships on adolescent GD, with particular emphasis on the role of teacher−student conflict. Adverse teacher−student relationships fail to fulfill the fundamental psychological needs of adolescents and undermine individual feelings of security (19), consequently heightening the risk of GD (36). If adolescents struggle to establish a proper achievement goal orientation, their focus may shift from the school environment to online games (20). Hence, we assume that aiding adolescents in effectively navigating their relationships with teachers is of equal importance and has been overlooked previously compared with peer and parent−child relationships.

Additionally, among the three nodes most closely associated with GD, the strength centrality of parent–child conflict is significantly weaker than that of peer fear and inferiority and teacher–student conflict, indicating that, in comparison to teachers and peers, parents’ impact on adolescent GD behavior is less pronounced and more distant. This can be attributed to the fact that during adolescence, individuals progressively spend less time with their parents, leading to a gradual decrease in parental influence on individuals. Viewed through the lens of developmental psychology, adolescents undergo a process of emotional, behavioral, and perspective separation from their parents. During this period, adolescents within the school environment spend more time with peers and teachers than with parents. Hence, in contrast to parent−child relationships, peer relationships and teacher−student relationships exert a more substantial impact on GD. Researchers have affirmed that adolescent peer attachment rather than parent–child relationships is negatively correlated with individuals’ GD behavior (21, 23).

One notable finding was that in the network analysis model, compared to the components of conflict and rejection, the positive components symbolizing companionship and warmth in three types of interpersonal relationships have a weaker association with GD (for example, the association between GD and parent−child companionship, parent−child instrumental aid, parent−child support, peer acceptance, teacher−student intimacy). Some components failed to exhibit the expected negative correlation, including parent−child intimacy, teacher−student satisfaction, teacher−student support, and teacher−student conflict. This contradicts the widely held view that good interpersonal relationships may serve as a protective factor against adolescents’ GD (37). The possible reasons is that adolescents’ gaming attitudes are significantly influenced by their social environment. Parents’ leniency and lack of regulation regarding online gaming may unintentionally foster a permissive attitude, increasing adolescents’ risk of gaming disorder (GD) (38). According to Wu et al. (37), peers’ approval of gaming is closely linked to the severity of adolescents’ GD. Furthermore, the relationship between interpersonal relationships and GD is complex, involving complex interactions with multiple social factors. Although typically protective, the influence of positive relationships on GD can be eclipsed by peer pressure and the specific dynamics of the gaming community.

These results indicate that the interplay between interpersonal relationships and GD is primarily characterized by the negative correlation of interpersonal relationships with GD, which supports the social compensation hypothesis that individuals lacking real-life social support may seek comfort on the internet (39). Adolescents who are struggling with interpersonal conflicts are more likely to turn to online games as a means of escaping the negative emotions that arise from these conflicts in an attempt to compensate for the lack of authentic social interaction within the gaming environment. Moreover, our findings extend the social compensation hypothesis by revealing the different effects of various interpersonal relationships on GD among adolescents. Specifically, the potential for social compensation through gaming is most pronounced in adolescents with abnormal peer relationships. This suggests that the presence of strained or dysfunctional peer dynamics may drive adolescents to seek alternative avenues for social engagement and validation, and online gaming can serve as a platform that offers a sense of community and belonging that may be lacking in their offline lives.

The findings suggest a negative correlation between interpersonal relationships and GD, corroborating the social compensation hypothesis; individuals deficient in real-life social support may turn to the internet for solace (39). Based on the social compensation hypothesis, individuals with impaired real-life social relationships might enhance their online social engagement to offset these shortcomings. Adolescents confronting real-world interpersonal conflicts could view in-game social interactions as more secure and comforting; thus, they tend to form social bonds with virtual companions in games to compensate for the absence of authentic social interaction in their actual environment. Furthermore, our research expands upon the social compensation hypothesis, by elucidating the distinct effects of diverse interpersonal dynamics on adolescent gaming disorder (GD). Specifically, the hypothesis underscores the profound impact of interpersonal challenges on gaming addiction, yet the varying effects of these relationships on adolescent gaming addiction are not well understood. This study reveals that the propensity for social compensation through gaming is particularly evident in adolescents experiencing peer conflicts. This suggests that adolescents with strained or impaired peer relationships might turn to gaming as an alternative to real-world social engagement, as online games provide a sense of community and belonging absent in their offline lives.

Overall, our study enhances the understanding of how the three types of interpersonal relationships—specifically, the dimensions of conflict and rejection—are closely associated with GD among adolescents, with peer relationship abnormalities being particularly likely to precipitate GD behaviors. However, limitations of the study should be taken into account when considering the findings. Initially, this study did not prioritize age-related variations in interpersonal relationships and gaming disorders, hence no age data were collected from participants. Consequently, aligning with similar Chinese studies, the study exclusively involved adolescents from middle and high schools. Nonetheless, the omission of age data could impede a comprehensive analysis of the link between interpersonal dynamics and gaming disorders. Future studies should consider collecting age data to explore how interpersonal relationships correlate with GD across different adolescent age groups, thereby improving the findings’ applicability. Second, the cross-sectional design of the survey restricts the applicability of the results to diverse populations. Longitudinal data collection in future studies could elucidate the causal relationships and interactions between interpersonal relationships and GD.Third, our study utilized a nonclinical sample. However, the association between interpersonal relationships and GD may manifest differently in clinical populations, such as those diagnosed with severe gaming addiction. Therefore, future research could compare our results with clinical populations to explore these potential differences.

## 5 Conclusion

This study explored the associations between adolescent interpersonal relationships (parent−child, teacher−student, and peer relationships) and GD within a network model to evaluate the significance of these three types of relationships in GD. The findings indicate that conflict and rejection dimensions in all three types of interpersonal relationships are strongly linked to GD. Notably, peer fear and inferiority are most closely associated with GD, followed by teacher−student conflict. Parent–child conflict has a considerably weaker impact on GD than does the first two. These results suggest that interpersonal conflict is a risk factor for GD in adolescents. Interventions addressing interpersonal issues in adolescents may benefit individuals struggling with addiction in games.

## Data Availability

The raw data supporting the conclusions of this article will be made available by the authors, without undue reservation.
